# Supplementary material for: Network-Based Isoform Quantification with RNA-Seq Data for Cancer Transcriptome Analysis
Source: PLoS Comput Biol. 2015 Dec 23;11(12):e1004465. doi: 10.1371/journal.pcbi.1004465 (PMC4689380; doi:10.1371/journal.pcbi.1004465)
Supplement: S1 Text — (PDF) [file pcbi.1004465.s001.pdf]

Alternative model by network-based regularization

We introduce a network-based regularization to the base model as an alternative model and evaluate the probabilities of a read being generated by the transcripts in all the genes simultaneously as follows,

$$\mathcal{L}_{pen}(\boldsymbol{P};\boldsymbol{r}) = log(\mathcal{L}(\boldsymbol{P};\boldsymbol{r})) - \lambda\|\boldsymbol{AP} - \boldsymbol{WP}\|_2^2. \tag{1}$$

The term $\lambda\|\boldsymbol{AP} - \boldsymbol{WP}\|_2^2$ in equation (1) is a network constraint to encode prior knowledge from the transcript network. Given a transcript interaction network, we assume that the connected transcripts are more likely to co-express by introducing the following cost term over the expression $\boldsymbol{\pi}$,

$$\begin{aligned}
\Psi(\boldsymbol{P},\boldsymbol{\pi}) &= \sum_{i=1}^{|\boldsymbol{T}|}\left(\pi_i - \sum_{j\in \boldsymbol{nb}(i)} \frac{\pi_j}{|\boldsymbol{nb}(i)|}\right)^2 \\
&= \sum_{i=1}^{|\boldsymbol{T}|}\left(\frac{p_i|\boldsymbol{r}_{g(i)}|}{l_i} - \sum_{j\in \boldsymbol{nb}(i)} \frac{p_j|\boldsymbol{r}_{g(j)}|}{|\boldsymbol{nb}(i)|l_j}\right)^2 \\
&= \sum_{i=1}^{|\boldsymbol{T}|}\left(A_{ii}p_i - \sum_{j\in \boldsymbol{nb}(i)} W_{ij}p_j\right)^2 \\
&= \|\boldsymbol{AP} - \boldsymbol{WP}\|_2^2,
\end{aligned} \tag{2}$$

where $\boldsymbol{nb}(i)$ are the neighbors of transcript i. $g(i)$ and $g(j)$ are the genes containing transcripts i and j, respectively. $|\boldsymbol{r}_{g(i)}|$ denotes the number of reads aligned to gene $g(i)$. l_i and l_j are the length of transcripts i and j. $\boldsymbol{A}$ is a diagonal matrix, where $A_{ii} = |\boldsymbol{r}_{g(i)}|/l_i$. $\boldsymbol{W}$ contains the weights of transcript pairs in the transcript network, where $W_{ij} = |\boldsymbol{r}_{g(j)}|/(|\boldsymbol{nb}(i)|l_j)$. Minimizing $\Psi(\boldsymbol{P},\boldsymbol{\pi})$ ensures that each transcript will receive an expression close to the average expression of its neighbors in the transcript network. To solve equation (1) we used CVX, a package for specifying and solving convex programs [1, 2]. The framework estimates the expressions of transcripts in all the genes together in one optimization. We applied this framework to the small network with 898 transcripts on MCF7 breast cancer cell line RNA-Seq data. Overall, the results between Net-RSTQ and the alternative framework can be highly similar as shown in S8 Table when parameter λs are tuned. However, the algorithm converges slowly (S4 Figure) compared to the convergence of Net-RSTQ in Figure 7 in the main manuscript). It is clear that the alternative model does not scale to larger networks.

References

1. Grant M, Boyd S (2014). CVX: Matlab software for disciplined convex programming, version 2.1. `http://cvxr.com/cvx`.

2. Grant M, Boyd S (2008) Graph implementations for nonsmooth convex programs. In: Blondel V, Boyd S, Kimura H, editors, Recent Advances in Learning and Control, Springer-Verlag Limited, Lecture Notes in Control and Information Sciences. pp. 95–110. `http://stanford.edu/~boyd/graph_dcp.html`.
