# Supplementary material for: Network-Based Isoform Quantification with RNA-Seq Data for Cancer Transcriptome Analysis
Source: PLoS Comput Biol. 2015 Dec 23;11(12):e1004465. doi: 10.1371/journal.pcbi.1004465 (PMC4689380; doi:10.1371/journal.pcbi.1004465)
Supplement: S2 Text — (PDF) [file pcbi.1004465.s002.pdf]

Steps of generating the simulation data

Below are the steps of generating the simulation data,

1. Generate 397 gene expression E by sampling a poisson distribution.
2. For the genes with multiple transcripts, generate isoform expressions from a mixed power law by the flux-simulator to calculate the initial proportion $p_{ik}(0)$ for each transcript T_{ik}.
3. Let the initial isoform expression $\pi_{ik}(0) = E_i * p_{ik}(0)$ + gaussian noise
4. Let $\alpha = 1$; Repeat $p_{ik} = (\alpha * \sum_{j \in nb(T_{ik})} \frac{\pi_{g(j),j}}{|nb(T_{ik})|} + \pi_{ik}(0)) / \sum_q (\alpha * \sum_{j \in nb(T_{iq})} \frac{\pi_{g(j),j}}{|nb(T_{iq})|} + \pi_{iq}(0))$.
5. After convergence, $\pi_{ik} = E_i * p_{ik}$.
6. $\boldsymbol{\pi}$ is further normalized and used with flux-simulator as the ground truth expressions.
