# Supplementary material for: Network-Based Isoform Quantification with RNA-Seq Data for Cancer Transcriptome Analysis
Source: PLoS Comput Biol. 2015 Dec 23;11(12):e1004465. doi: 10.1371/journal.pcbi.1004465 (PMC4689380; doi:10.1371/journal.pcbi.1004465)
Supplement: S3 Text — (PDF) [file pcbi.1004465.s003.pdf]

Cufflinks and RSEM command line

Cufflinks 2.1.1[1] was applied to generate isoform expression as one of the baseline to compare with Net-RSTQ using the following command:

./cufflinks -p 4 -F 0 -G hg19RefSeq.gtf -o x.bam.

RSEM1.2.20[2] was applied to generate isoform expression as another baseline to compare with Net-RSTQ in simulation and qRT-PCR studies using the following command:

./rsem-calculate-expression –paired-end –bowtie2 –bowtie2-path bowtie2/ -p 2 x1.fastq x2.fastq hg19RefSeq x.

References

1. Trapnell C, Williams BA, Pertea G, Mortazavi A, Kwan G, et al. (2010) Transcript assembly and quantification by RNA-Seq reveals unannotated transcripts and isoform switching during cell differentiation. Nat Biotech 28: 511-515.

2. Li B, Dewey CN (2011) RSEM: accurate transcript quantification from RNA-Seq data with or without a reference genome. BMC Bioinformatics 12: 323.
