# Supplementary figures and images for: Network-Based Isoform Quantification with RNA-Seq Data for Cancer Transcriptome Analysis
Source: PLoS Comput Biol. 2015 Dec 23;11(12):e1004465. doi: 10.1371/journal.pcbi.1004465 (PMC4689380; doi:10.1371/journal.pcbi.1004465)

A

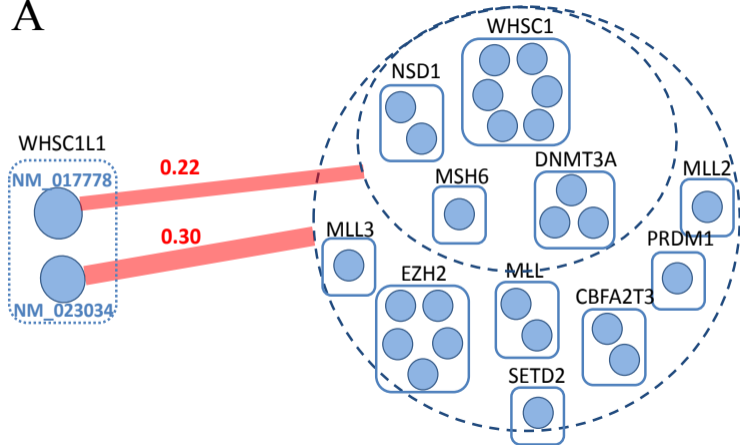

B

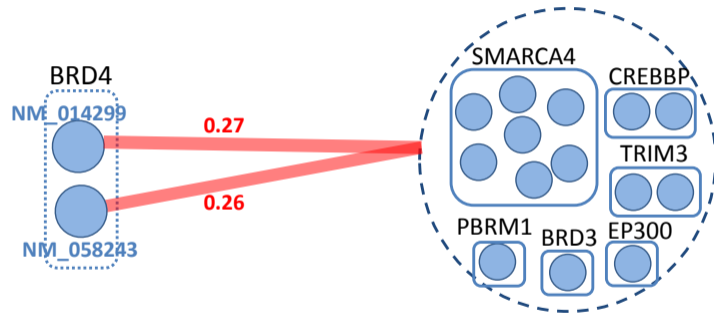

Supplement: S1 Fig — (A) Transcripts in WHSC1L1 with correlation coefficients calculated on the OV dataset. (B) Transcripts in BRD4 with correlation coefficients calculated on the BRCA dataset. Both examples are shown with the neighbors in the small transcript network. (PDF) [file pcbi.1004465.s004.pdf]

A

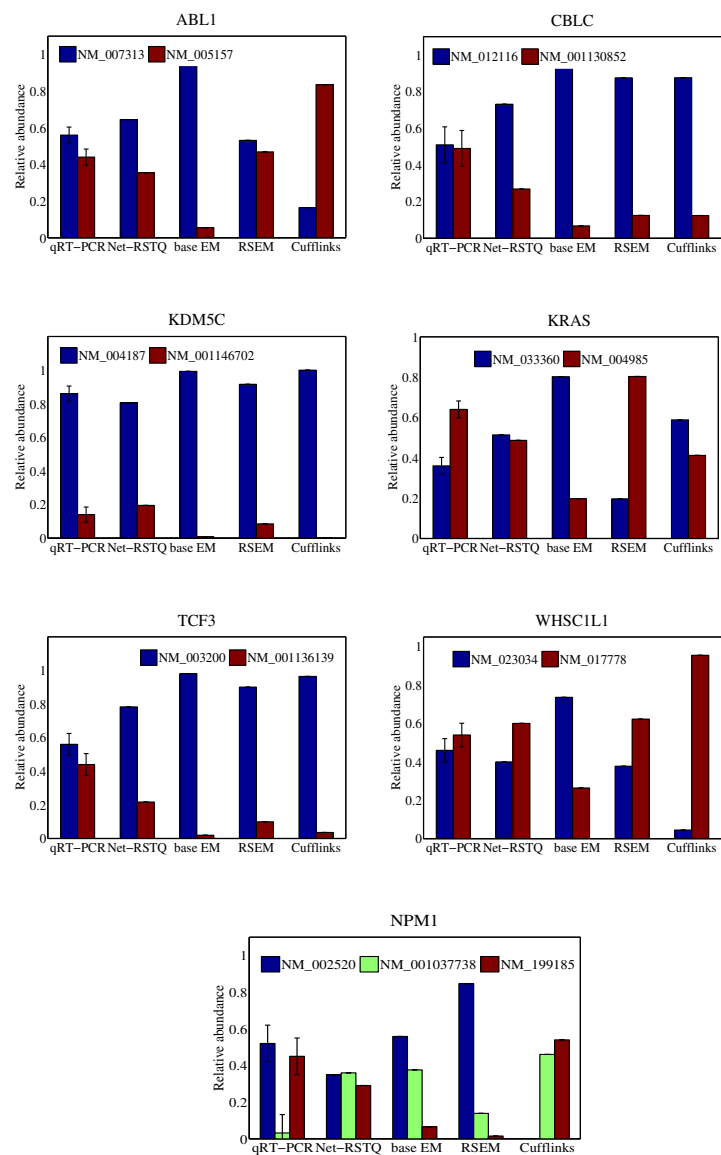

B

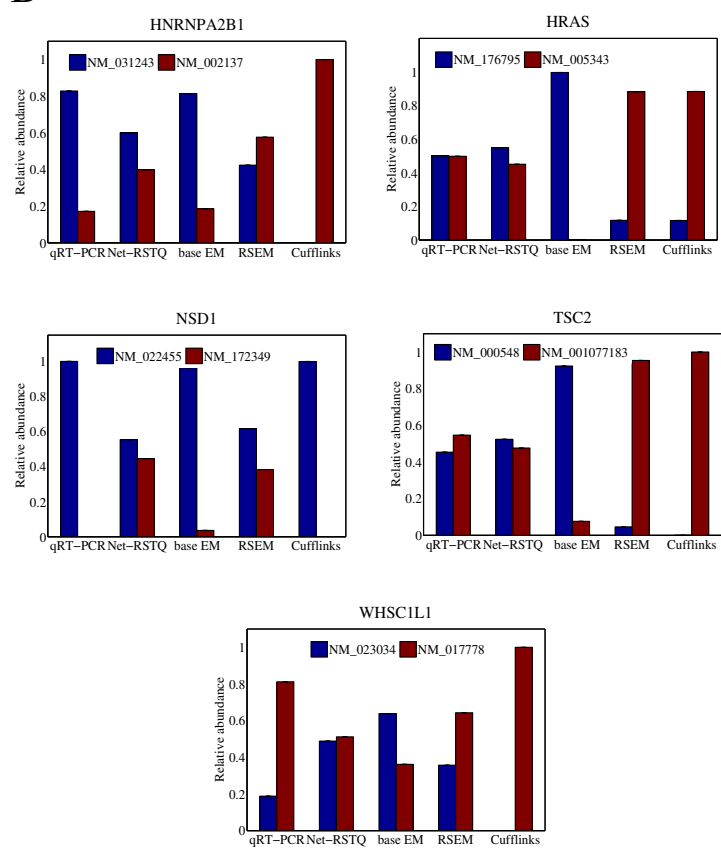

C

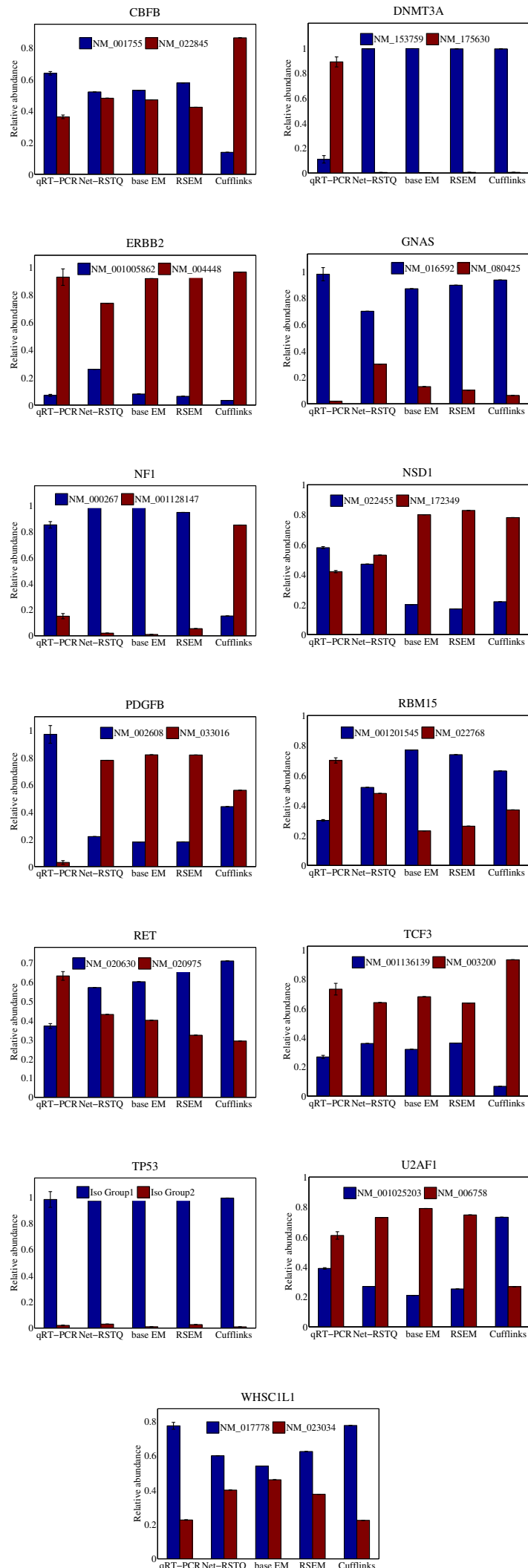

Supplement: S2 Fig — The relative abundance of the transcripts in 7 tested genes in H9 stem cell line (A), 5 tested genes in OVCAR8 ovarian cancer cell line (B), and 13 tested genes in MCF7 breast cancer cell line (C) estimated by Net-RSTQ, base EM, Cufflinks and RSEM was compared with the qRT-PCR experiments. The total abundance is normalized to 1 over the measured transcripts in each gene. (PDF) [file pcbi.1004465.s005.pdf]

**A**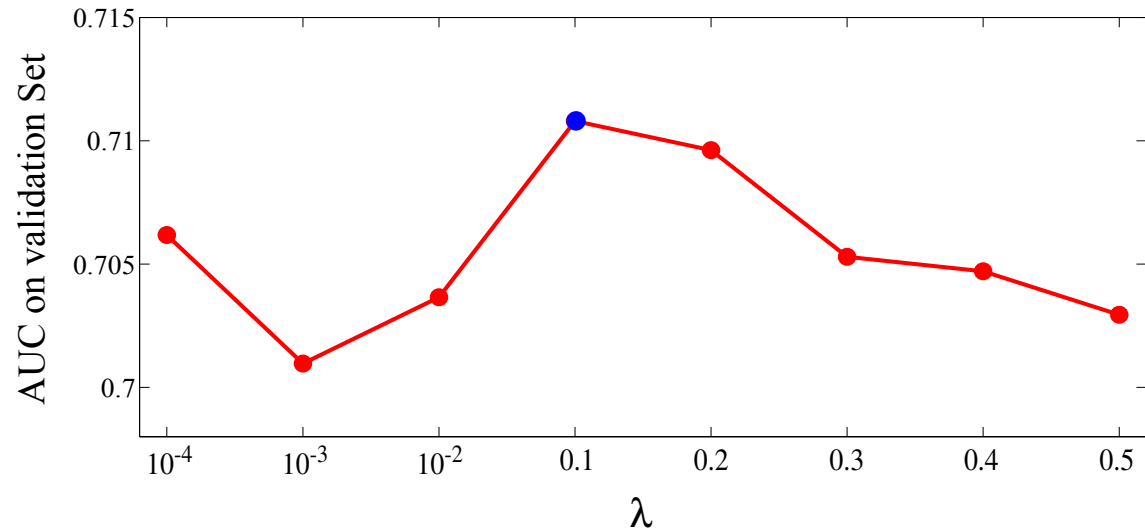**B**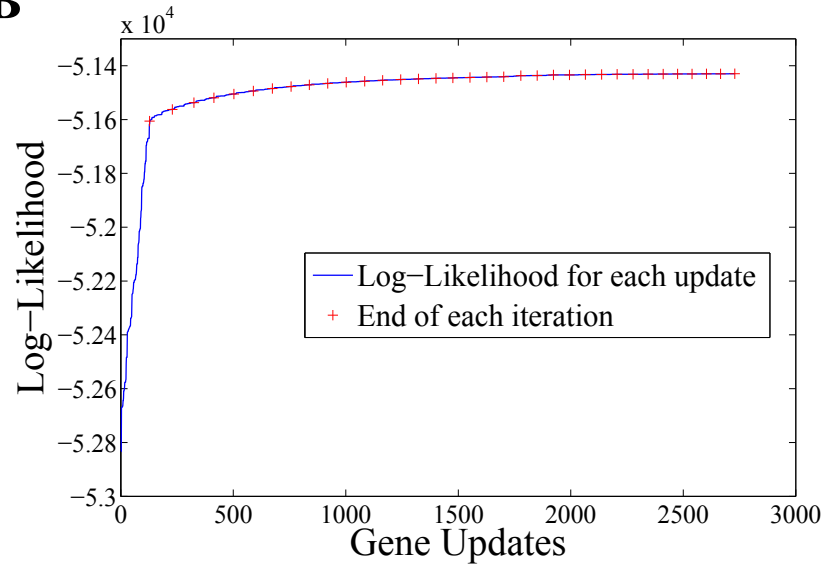

Supplement: S3 Fig — The experiment done on BRCA (survival) dataset. (A) Effect of varying λ on the classification performance. The plot shows the average AUC learned from the 100 repeats on validation set for different λs with the optimal λ in blue. (B) Convergence analysis by the total log-likelihood. The plot shows the change of total log-likelihood in Net-RSTQ with each gene update. Each red cross indicates the end of each round t in line 2 of Algorithm 1. (PDF) [file pcbi.1004465.s006.pdf]

**A**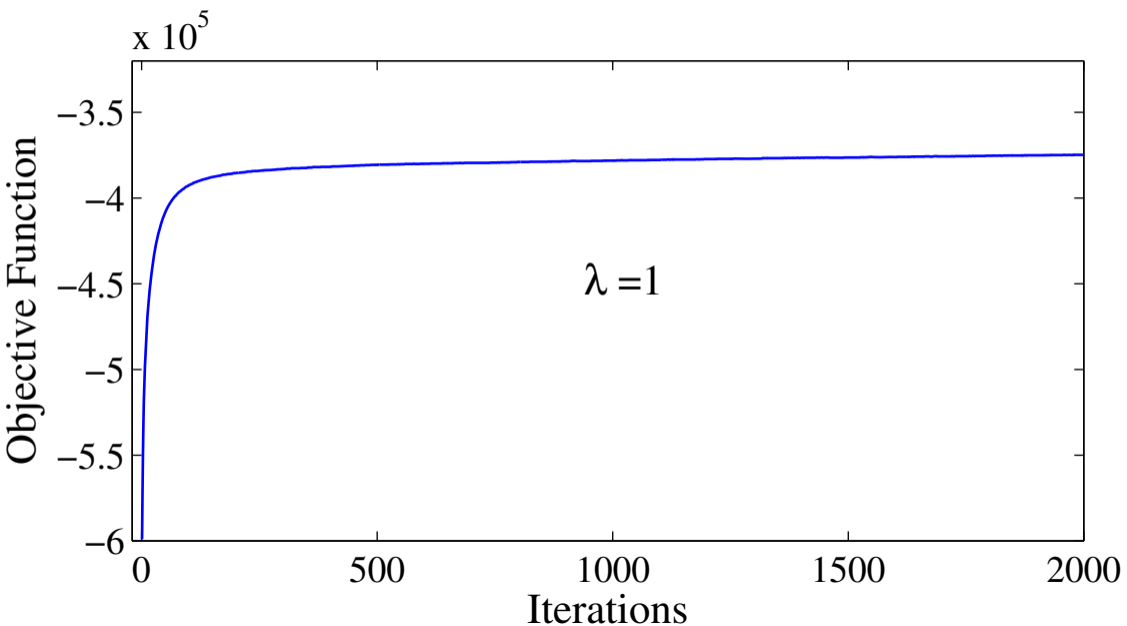**B**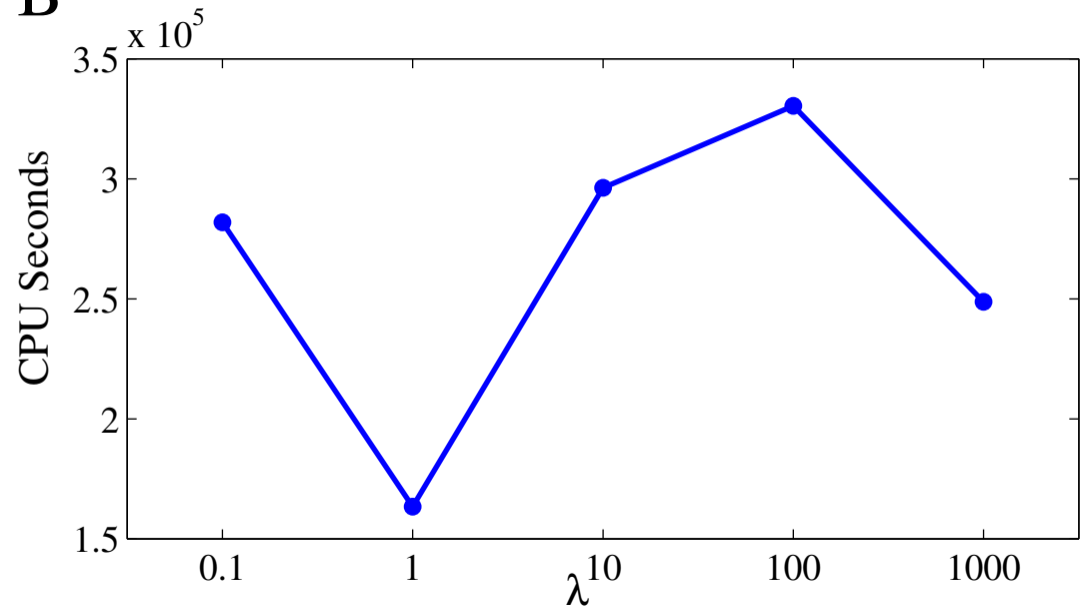

Supplement: S4 Fig — (PDF) [file pcbi.1004465.s007.pdf]
