# Supplementary material for: Network-Based Isoform Quantification with RNA-Seq Data for Cancer Transcriptome Analysis
Source: PLoS Comput Biol. 2015 Dec 23;11(12):e1004465. doi: 10.1371/journal.pcbi.1004465 (PMC4689380; doi:10.1371/journal.pcbi.1004465)
Supplement: S1 Table — * The numbers refer to the isoforms in the first column. (PDF) [file pcbi.1004465.s008.pdf]

Gene/Transcript(isoform) Names	Primer Names*	Forward	Reverse
ABL1	Template 1&2	5-GGTTGGTGACTTCCACAGGAAA	5-CACCGTCAGGCTGTATTTCTTCC
NM_007313(iso2)	qPCR 1&2	5-TGAAAAGCTCCGGGTCTTAGG	5-TTGACTGGCGTGATGTAGTTG
NM_005157(iso1)	qPCR 2	5-TAGCCAAAGACCATCAGCGTT	5-TTCGCGGTTATCAATTTCATGT
CBCL	Template 1&2	5-ACCCTGTGGAACCAGGCTGC	5-CACCTGCCCCAGCTCCAACT
NM_012116(iso1)	qPCR 1&2	5-ACCACCATTGACCTCACCTGC	5-ACTGCCAGGAGCTGCCAGTT
NM_001130852(iso2)	qPCR 1	5-CATCCTGCAGACCATCCCTG	5-GGCCGAGCTCAGTCAGGTCT
KDM5C	Template 1&2	5-GACCTGCTCGAGGTGACCCT	5-AAGCTTTCTTCAGATCACAGGGAG
NM_004187(iso1)	qPCR 1&2	5-GCCTCTAACCAGCATTCCCA	5-TCTCTGGAATGGTGATGGCC
NM_001146702(iso2)	qPCR 1	5-AGAGGCTGAGGAGGTCCAGG	5-CCAAGCCATTCTGGTTCTCC
TCF3	Template 1&2	5-TGAATCCCAAAGCAGCCTG	5-TCTTGTAACTAATGTTTTTATTTTCCTTA
NM_003200(iso1)	qPCR 1&2	5-TGAATCCCAAAGCAGCCTGT	5-GGTTGTGGGCTTCGCTCAG
NM_001136139(iso2)	qPCR 1	5-GTATGCCTCCGTGGGACGA	5-GGAGCTCCTGGACCCAGTGT
WHSC1L1	Template 1	5-CAGTTCCTCAGGCTACAGTGAAGA	5-CATACAACAAACAGACATCTAGATCAAC
NM_023034(iso1)	Template 2	5-CAGTTCCTCAGGCTACAGTGAAGA	5-GTAATGTAGTTTCTTGCCAGCTTTACA
NM_017778(iso2)	qPCR 1	5-GTCGGCGGCTTGATAAACAGT	5-GTACCCATCCAGCTCAAACCG
	qPCR 2	5- CCCTTCAGCTACTGCAGATGC	5-CCAGGCACTCCAGGTGAAAGT
KRAS	Template 1	5-TTCCTGCTCCATGCAGACTGT	5-TAAGAAGTAATCAACTGCATGCACCA
NM_033360(iso2)	Template 2	5-TACATTGGTGAGGGAGATCCGA	5-TAAGAAGTAATCAACTGCATGCACCA
NM_004985(iso1)	qPCR 1	5-TTCCTGCTCCATGCAGACTGT	5-GCACCAAAAACCCCAAGACAG
	qPCR 2	5- TACATTGGTGAGGGAGATCCGA	5-TAGAAGGCATCATCAACACCCA
NMP1	Template 1&2&3	5-TCCTTTCCCTGGTGTGATTCC	5-CATTGTCAGGTGAGGCAAATGC
NM_002520(iso1)	qPCR 1&2&3	5-TCCTTTCCCTGGTGTGATTCC	5-TCGGCCTTTAGTTCACAACCG
NM_0010337738(iso3)	qPCR 1&3	5-AGCTGAAGAAAAAGCGCCAGT	5-CTTTTGTGCATTTTTGGCTGG
NM_199185(iso2)	qPCR 3	5-AAGCCCAAAGATGGGGAGAA	5-AAGGGCAAGGTTCACTGAATCA

S1 Table. Primer sets of the transcripts in seven genes of H9 stem cell line. * The numbers refer to the isoforms in the first column.
