# Supplementary material for: Network-Based Isoform Quantification with RNA-Seq Data for Cancer Transcriptome Analysis
Source: PLoS Comput Biol. 2015 Dec 23;11(12):e1004465. doi: 10.1371/journal.pcbi.1004465 (PMC4689380; doi:10.1371/journal.pcbi.1004465)
Supplement: S2 Table — * Gene contains more transcript(s) which can not be quantified by qRT-PCR. (PDF) [file pcbi.1004465.s009.pdf]

Gene Name	Transcript Name	Primer Sequence - Forward	Primer Sequence - Reverse
HNRNPA2B1	NM_031243	TCC GCG ATG GAG GAA AAC TTT AG	GCC ACC AAT AAA GAG CTT ACG G
	NM_002137	AGC GGC AGT TCT CAC TAC AG	TCC TTT TCT CTC CTC CAT CG
HRAS*	NM_176795	CCG CTC TGG CTC TAG CTC	ACC AAC GTG TAG AAG GCA TCC
	NM_005343	AGG ATG CCT TCT ACA CGT TGG	CAT GTC CTG AGC TTG TGC CT
NSD-1	NM_022455	TCG CCA TTC TTG CCA TTA GC	TTT TCA TTG CTG CCG TCC AC
	NM_172349	ATT GTC TGC TGC CCT TTT CC	TGG AAT CTG GAT CAT CCC GA
TSC2*	NM_000548	CTC TCC ACC CGT GAA AGA ATT C	GAC CAC ATG TTC AGA CAC ACT G
	NM_001077183	AAC GAG AGA CCC AAG AGG AT	GA CGT ATC GAG CCA TCA TGT C
WHSC1L1	NM_023034	ATG TAA AAC TGG GGC AGC AC	AAG CAC CAA CAG AAC AAC GC
	NM_017778	TTT CGG TTT GAG CTG GAT GG	TTT GGG CTG TTT GGC AAA CC

S2 Table. Primer sets of the transcripts in five genes of OVCAR8 cancer cell line. * Gene contains more transcript(s) which can not be quantified by qRT-PCR.
