# Supplementary material for: Network-Based Isoform Quantification with RNA-Seq Data for Cancer Transcriptome Analysis
Source: PLoS Comput Biol. 2015 Dec 23;11(12):e1004465. doi: 10.1371/journal.pcbi.1004465 (PMC4689380; doi:10.1371/journal.pcbi.1004465)
Supplement: S3 Table — * Gene contains more transcript(s) which can not be quantified by qRT-PCR. (PDF) [file pcbi.1004465.s010.pdf]

Gene Name	Transcript Name	Primer Sequence - Forward	Primer Sequence - Reverse
ERBB2	NM_001005862	5-CACAGATAAAACGGGGGCAC	5-CAGGGTCTGAGTCTCTGTGCT
	NM_004448	5-GAGGGCTGCTTGAGGAAGTAT	5-TTTCTCCGGTCCCAATGGAG
NSD1	NM_022455	5-GACACGGTGCAGTCAAATCG	5-GCTGCCGTCCACTTCATTTC
	NM_172349	5-AGAAGAAATTGTCTGCTGCCC	5-GGATCATCCGAAAGGGCTGT
U2AF1*	NM_001025203	5-TTGGAGCATGTCGTCATGGAG	5-CTGTGCACTGTTTTGGGGATT
	NM_006758	5-TGCCCTCTTGAACATTTACCGT	5-CTGCATCTCCACATCGCTCA
PDGFB	NM_002608	5-CTCCGCGCTTTCCGATTTTG	5-AGAGGAAAAGGAACACGGCA
	NM_033016	5-GACTGAGCAGGAATGGTGAGAT	5-TCAAAGGAGCGGATCGAGTG
DNMT3A*	NM_153759	5-GCAGCTACTTCCAGAGCTTCA	5-TTTCAGGCTACGATCCACGC
	NM_175630	5-GGGCAGCAGATACCCTGTTT	5-GGCTGGGCAGTACACAGAAT
GNAS*	NM_016592	5-CGAGTCTTAGGCTGCGGAAT	5-GCACCTACCTTCCTGACCAC
	NM_080425	5-CACTCCCGTCAACATGGACA	5-GTACCCCGGAGAGGGTACTT
RBM15	NM_001201545	5-ATGCCTTCCCACCTTGTGAG	5-TCAACCAGTTTTGCACGGAC
	NM_022768	5-AACAAGAAGAGAGAAAACTTGGCG	5-TTTCCTCCCTTTAGGGACACC
RET	NM_020630	5-TGCCCAGCAACTTAGGATGG	5-TTGATTCCCACCCCAGAAGC
	NM_020975	5-AATGGAAAGTCTACCGGCCC	5-CAGAGCTCTTACCCGGTGTG
TCF3	NM_001136139	5-GAGAAAGACCTGAGGGACCG	5-GGCCTCGTTAATATCCCGCA
	NM_003200	5-CAACTGCACCTCAACAGCGA	5-CTCCAAGTTCAGGATGACCGA
WHSC1L1	NM_017778	5-GCCTCTCAGTACAGCACTCC	5-GCCTGCCCATGTTAATGCTG
	NM_023034	5-AGAAAGGTGCCAGCGAGATT	5-GCAGGTCACTCAGTCCTCTA
CBFB	NM_001755	5-GGATGCATTAGCACAACAGGC	5-GCCAGCAGCTGTGAAACTCT
	NM_022845	5-CGGGAGGAAATGGAGGCAAG	5-GTAAAGATGGGCAGCACACAT
TP53	Iso Group1	5-GATGAAGCTCCCAGAATGCC	5-GTAGCTGCCCTGGTAGGTTT
	Iso Group2	5-GAGGTGTAGACGCCAACTCT	5-AAGTCAGGGCACAAGTGAACA
NF1*	NM_000267	5-TGAGGAAAACCAGCGGAACC	5-GCTGGCTAACCACCTGGTATAAA
	NM_001128147	5-GTGGAATCCTGATGCTCCTGT	5-AAAACCATAAAACCTTTGGAAGTGT

S3 Table. Primer sets of the transcripts in thirteen genes of MCF7 cancer cell line. * Gene contains more transcript(s) which can not be quantified by qRT-PCR.
