# Supplementary material for: Network-Based Isoform Quantification with RNA-Seq Data for Cancer Transcriptome Analysis
Source: PLoS Comput Biol. 2015 Dec 23;11(12):e1004465. doi: 10.1371/journal.pcbi.1004465 (PMC4689380; doi:10.1371/journal.pcbi.1004465)
Supplement: S4 Table — We consider the subnetwork of genes that are members of one KEGG pathway and calculated the density of DDIs in the subnetwork. (PDF) [file pcbi.1004465.s011.pdf]

KEGG Pathway	# of gene in KEGG	# of overlapped gene	Interactions	Density
Long-term potentiation	67	30	552	61.33%
Fc epsilon RI signaling pathway	70	51	1426	54.83%
Fc gamma R-mediated phagocytosis	91	45	1002	49.48%
Axon guidance	127	56	1526	48.66%
VEGF signaling pathway	61	39	736	48.39%
GnRH signaling pathway	92	53	1356	48.27%
Phosphatidylinositol signaling system	82	26	318	47.04%
ErbB signaling pathway	87	70	2272	46.37%
Inositol phosphate metabolism	63	14	86	43.88%
Neuroactive ligand-receptor interaction	275	38	626	43.35%
Glycerophospholipid metabolism	95	12	62	43.06%
Olfactory transduction	407	15	92	40.89%
Neurotrophin signaling pathway	120	88	3128	40.39%
Central carbon metabolism in cancer	67	38	580	40.17%
Natural killer cell mediated cytotoxicity	134	47	848	38.39%
Glutamatergic synapse	116	21	168	38.10%
Retrograde endocannabinoid signaling	103	25	238	38.08%
RNA polymerase	32	11	46	38.02%
Glioma	65	46	800	37.81%
Ras signaling pathway	228	119	5182	36.59%
Inflammatory mediator regulation of TRP channels	99	45	722	35.65%
Long-term depression	60	25	220	35.20%
Gap junction	89	42	620	35.15%
B cell receptor signaling pathway	72	45	706	34.86%
Tight junction	138	49	830	34.57%
Glycerolipid metabolism	59	13	58	34.32%
mTOR signaling pathway	60	35	418	34.12%
T cell receptor signaling pathway	104	80	2178	34.03%
Vascular smooth muscle contraction	121	43	622	33.64%
Non-small cell lung cancer	56	45	662	32.69%
Chemokine signaling pathway	189	86	2408	32.56%
Cell adhesion molecules (CAMs)	142	32	332	32.42%
Choline metabolism in cancer	101	56	1012	32.27%
MAPK signaling pathway	257	156	7830	32.17%
Epithelial cell signaling in Helicobacter pylori infection	68	36	414	31.94%
Oxytocin signaling pathway	159	63	1264	31.85%
Type II diabetes mellitus	48	23	166	31.38%
Adherens junction	73	48	722	31.34%
Lysine degradation	52	11	36	29.75%
Circadian entrainment	97	28	232	29.59%
Prolactin signaling pathway	72	52	792	29.29%
Insulin signaling pathway	140	75	1634	29.05%
Cholinergic synapse	113	42	512	29.02%
Morphine addiction	93	21	126	28.57%
Bladder cancer	38	34	322	27.85%
Platelet activation	131	57	892	27.45%
Progesterone-mediated oocyte maturation	88	63	1074	27.06%
Toll-like receptor signaling pathway	106	56	842	26.85%
Rap1 signaling pathway	211	119	3722	26.28%
Leukocyte transendothelial migration	118	51	680	26.14%
Pancreatic cancer	66	58	870	25.86%
Complement and coagulation cascades	69	23	136	25.71%
Acute myeloid leukemia	57	48	586	25.43%
Renal cell carcinoma	66	55	754	24.93%
Endometrial cancer	52	41	414	24.63%
Sphingolipid signaling pathway	120	66	1068	24.52%
Focal adhesion	207	103	2600	24.51%
Regulation of actin cytoskeleton	215	95	2210	24.49%
FoxO signaling pathway	134	94	2102	23.79%
Amyotrophic lateral sclerosis (ALS)	51	20	94	23.50%
Osteoclast differentiation	131	82	1544	22.96%
Melanoma	71	50	566	22.64%
Serotonergic synapse	114	29	190	22.59%
Gastric acid secretion	75	24	130	22.57%
Hepatitis C	133	73	1194	22.41%
Dopaminergic synapse	131	44	426	22.00%
Calcium signaling pathway	180	51	570	21.91%
Pathogenic Escherichia coli infection	55	22	104	21.49%
Influenza A	175	76	1172	20.29%
Estrogen signaling pathway	100	51	524	20.15%
Chronic myeloid leukemia	73	63	796	20.06%
Aldosterone-regulated sodium reabsorption	39	22	96	19.83%
Ovarian steroidogenesis	51	18	64	19.75%
NOD-like receptor signaling pathway	57	31	186	19.35%
cAMP signaling pathway	200	82	1298	19.30%
Colorectal cancer	62	52	498	18.42%
Proteoglycans in cancer	204	130	3082	18.24%
Prostate cancer	89	70	884	18.04%
RIG-I-like receptor signaling pathway	70	35	220	17.96%
Signaling pathways regulating pluripotency of stem cells	142	84	1250	17.72%
cGMP-PKG signaling pathway	167	56	542	17.28%
Thyroid hormone signaling pathway	119	62	662	17.22%
TGF-beta signaling pathway	80	51	446	17.15%
Endocytosis	213	84	1204	17.06%
ECM-receptor interaction	87	23	90	17.01%
Adipocytokine signaling pathway	70	38	242	16.76%
Hepatitis B	146	98	1510	15.72%
Jak-STAT signaling pathway	156	69	734	15.42%
HIF-1 signaling pathway	103	68	710	15.35%
Glucagon signaling pathway	102	33	166	15.24%
Ubiquitin mediated proteolysis	137	66	654	15.01%
TNF signaling pathway	110	79	894	14.32%
PI3K-Akt signaling pathway	347	166	3904	14.17%
AMPK signaling pathway	124	59	476	13.67%
Wnt signaling pathway	140	76	774	13.40%
Adrenergic signaling in cardiomyocytes	149	62	496	12.90%
NF-kappa B signaling pathway	91	55	380	12.56%
MicroRNAs in cancer	297	109	1484	12.49%
Cell cycle	124	91	734	8.86%
Pathways in cancer	398	260	5702	8.43%

S4 Table. Overlapped KEGG pathways with large transcript network. We consider the subnetwork of genes that are members of one KEGG pathway and calculated the density of DDIs in the subnetwork.
