# Supplementary material for: Network-Based Isoform Quantification with RNA-Seq Data for Cancer Transcriptome Analysis
Source: PLoS Comput Biol. 2015 Dec 23;11(12):e1004465. doi: 10.1371/journal.pcbi.1004465 (PMC4689380; doi:10.1371/journal.pcbi.1004465)
Supplement: S5 Table — * Standard deviation of Iso1 + Iso3 is 5.7% and Iso3 is 4.4% (PDF) [file pcbi.1004465.s012.pdf]

Gene Name	Transcript Name	Estimated Proportion				qRT-PCR Results
		Net-RSTQ	base EM	Cufflinks	RSEM	
ABL1	NM_007313	64.46%	94.45%	16.48%	53.14%	56±4.4%
	NM_005157	35.54%	5.55%	83.52%	46.86%	44±4.4%
CBLC	NM_012116	73.12%	93.23%	87.59%	87.54%	51±9.8%
	NM_001130852	26.88%	6.77%	12.41%	12.46%	49±9.8%
KDM5C	NM_004187	80.52%	99.22%	99.95%	91.52%	86±4.5%
	NM_001146702	19.48%	0.78%	0.05%	8.48%	14±4.5%
KRAS	NM_033360	51.36%	80.23%	58.82%	19.67%	36±4.2%
	NM_004985	48.64%	19.77%	41.18%	80.33%	64±4.2%
NPM1	NM_002520 (Iso1)	34.92%	55.84%	0%	84.62%	52%*
	NM_199185 (Iso2)	29.09%	6.56%	53.97%	1.52%	45%
	NM_001037738 (Iso3)	35.99%	37.60%	46.03%	13.86%	3.2%
TCF3	NM_003200	78.31%	98.11%	96.42%	90.06%	56±6.5%
	NM_001136139	21.69%	1.89%	3.58%	9.94%	44±6.5%
WHSC1L1	NM_023034	39.98%	73.61%	4.50%	37.72%	46±6.1%
	NM_017778	60.02%	26.39%	95.50%	62.28%	54±6.1%

S5 Table. qRT-PCR results on H9 stem cell line. * Standard deviation of Iso1+Iso3 is 5.7% and Iso3 is 4.4%
