# Supplementary material for: Network-Based Isoform Quantification with RNA-Seq Data for Cancer Transcriptome Analysis
Source: PLoS Comput Biol. 2015 Dec 23;11(12):e1004465. doi: 10.1371/journal.pcbi.1004465 (PMC4689380; doi:10.1371/journal.pcbi.1004465)
Supplement: S6 Table — * Gene contains more transcript which can not be quantified by qRT-PCR. (PDF) [file pcbi.1004465.s013.pdf]

Gene Name	Transcript Name	Estimated Proportion				qRT-PCR Results
		Net-RSTQ	base EM	Cufflinks	RSEM	
HNRNPA2B1	NM_031243	60.07%	81.42%	0%	42.37%	82.83%
	NM_002137	39.93%	18.58%	100%	57.63%	17.17%
HRAS*	NM_176795	54.92%	100%	11.62%	11.72%	50.16%
	NM_005343	45.08%	0%	88.38%	88.28%	49.84%
NSD1	NM_022455	55.40%	96.39%	99.94%	61.65%	99.98%
	NM_172349	44.60%	3.61%	0.06%	38.35%	0.02%
TSC2*	NM_000548	52.39%	92.36%	0.01%	4.62%	45.36%
	NM_001077183	47.61%	7.64%	99.99%	95.38%	54.64%
WHSC1L1	NM_023034	48.84%	63.85%	0.01%	35.69%	18.81%
	NM_017778	51.16%	36.15%	99.99%	64.31%	81.19%

S6 Table. qRT-PCR results on OVCAR8 cancer cell line. * Gene contains more transcript which can not be quantified by qRT-PCR.
