# Supplementary material for: Network-Based Isoform Quantification with RNA-Seq Data for Cancer Transcriptome Analysis
Source: PLoS Comput Biol. 2015 Dec 23;11(12):e1004465. doi: 10.1371/journal.pcbi.1004465 (PMC4689380; doi:10.1371/journal.pcbi.1004465)
Supplement: S7 Table — * Gene contains more transcript(s) which can not be quantified by qRT-PCR. (PDF) [file pcbi.1004465.s014.pdf]

Gene Name	Transcript Name	Estimated Proportion				qRT-PCR Results
		Net-RSTQ	base EM	Cufflinks	RSEM	
ERBB2	NM_001005862	26.16%	7.79%	3.35%	6.34%	7.35±0.75%
	NM_004448	73.84%	92.21%	96.65%	93.66%	92.65±6.0%
NSD1	NM_022455	46.77%	20.08%	21.94%	17.14%	58.34±0.70%
	NM_172349	53.23%	79.92%	78.06%	82.86%	41.66±0.75%
U2AF1*	NM_001025203	26.68%	21.11%	73.15%	25.33%	39.13±0.50%
	NM_006758	73.32%	78.89%	26.85%	74.67%	60.87±2.5%
PDGFB	NM_002608	21.51%	18.03%	43.93%	18.12%	97.69±6.5%
	NM_033016	78.49%	81.97%	56.07%	81.88%	2.31±1.4%
DNMT3A*	NM_153759	99.53%	99.77%	99.45%	99.46%	11.06±3.0%
	NM_175630	0.47%	0.23%	0.55%	0.54%	88.94±4.0%
GNAS*	NM_016592	69.65%	87.11%	93.62%	89.66%	98.27±5.0%
	NM_080425	30.35%	12.89%	6.38%	10.34%	1.73±0%
RBM15	NM_001201545	51.65%	77.37%	63.21%	73.85%	30.21±0.55%
	NM_022768	48.35%	22.63%	36.79%	26.15%	69.79±1.8%
RET	NM_020630	57.13%	60.36%	70.78%	67.76%	37.04±1.3%
	NM_020975	42.87%	39.64%	29.22%	32.24%	62.96±2.3%
TCF3	NM_001136139	35.90%	31.51%	6.70%	36.31%	26.85±1.1%
	NM_003200	64.10%	68.49%	93.30%	63.69%	73.15±4.0%
WHSC1L1	NM_017778	59.60%	54.38%	77.61%	62.43%	77.36±2.0%
	NM_023034	40.40%	45.62%	22.39%	37.57%	22.64±0.18%
CBFB	NM_001755	51.89%	52.80%	13.84%	57.67%	63.75±1.1%
	NM_022845	48.11%	47.20%	86.16%	42.33%	36.25±1.2%
TP53	Iso Group1	96.90%	99.37%	99.10%	97.41%	98.12±6.0%
	Iso Group2	3.10%	0.63%	0.90%	2.59%	1.88±0.30%
NF1*	NM_000267	98.08%	98.56%	15.14%	94.55%	85.76±2.5%
	NM_001128147	1.92%	1.44%	84.86%	5.45%	14.24±2.0%

S7 Table. qRT-PCR results on MCF7 cancer cell line. * Gene contains more transcript(s) which can not be quantified by qRT-PCR.
