# Supplementary material for: Network-Based Isoform Quantification with RNA-Seq Data for Cancer Transcriptome Analysis
Source: PLoS Comput Biol. 2015 Dec 23;11(12):e1004465. doi: 10.1371/journal.pcbi.1004465 (PMC4689380; doi:10.1371/journal.pcbi.1004465)
Supplement: S8 Table — The highest correlation coefficients for each λ in the alternative regularized framework is bold. (PDF) [file pcbi.1004465.s015.pdf]

	Regularized framework				
**Net-RSTQ**	λ	**0.1**	**1**	**10**	**100**
	**1e-04**	0.9689	0.9536	0.9442	0.8668
	**1e-03**	0.9696	0.9549	0.9453	0.8680
	**5e-03**	0.9722	0.9583	0.9488	0.8715
	**0.01**	0.9751	0.9620	0.9526	0.8751
	**0.05**	0.9887	0.9804	0.9720	0.8939
	**0.1**	0.9943	0.9895	0.9819	0.9038
	**0.2**	**0.9959**	0.9947	0.9882	0.9111
	**0.3**	0.9950	**0.9954**	0.9899	0.9141
	**0.4**	0.9938	0.9951	0.9905	0.9164
	**0.5**	0.9925	0.9945	0.9909	0.9188
	**0.6**	0.9910	0.9935	0.9912	0.9218
	**0.7**	0.9889	0.9920	**0.9914**	0.9258
	**0.8**	0.9854	0.9891	0.9911	0.9319
	**0.9**	0.9770	0.9815	0.9883	0.9424
	**0.99**	0.9328	0.9387	0.9543	**0.9601**
	**0.999**	0.9121	0.9181	0.9349	0.9578

S8 Table. Correlation Coefficients between the results of Net-RSTQ and the alternative regularized framework with different λs. The highest correlation coefficients for each λ in the alternative regularized framework is bold.
